# Human brain structure predicts individual differences in preconscious evaluation of facial dominance and trustworthiness

**DOI:** 10.1093/scan/nsu103

**Published:** 2014-09-04

**Authors:** Spas Getov, Ryota Kanai, Bahador Bahrami, Geraint Rees

**Affiliations:** ^1^Wellcome Trust Centre for Neuroimaging, Institute of Neurology, University College London, London, UK, ^2^UCL Institute of Cognitive Neuroscience, 17 Queen Square, London, UK, ^3^Sackler Centre for Consciousness Science, School of Psychology, University of Sussex, Pevensey 1, Brighton, UK, ^4^Interacting Minds Center, Aarhus University, and Centre of Functionally Integrative Neuroscience, Aarhus University, Aarhus, Denmark

**Keywords:** awareness, dominance, trustworthiness, voxel-based morphometry, continuous flash suppression

## Abstract

Social cues conveyed by the human face, such as eye gaze direction, are evaluated even before they are consciously perceived. While there is substantial individual variability in such evaluation, its neural basis is unknown. Here we asked whether individual differences in preconscious evaluation of social face traits were associated with local variability in brain structure. Adult human participants (*n* = 36) monocularly viewed faces varying in dominance and trustworthiness, which were suppressed from awareness by a dynamic noise pattern shown to the other eye. The time taken for faces to emerge from suppression and become visible (t2e) was used as a measure of potency in competing for visual awareness. Both dominant and untrustworthy faces resulted in slower t2e than neutral faces, with substantial individual variability in these effects. Individual differences in t2e were correlated with gray matter volume in right insula for dominant faces, and with gray matter volume in medial prefrontal cortex, right temporoparietal junction and bilateral fusiform face area for untrustworthy faces. Thus, individual differences in preconscious social processing can be predicted from local brain structure, and separable correlates for facial dominance and untrustworthiness suggest distinct mechanisms of preconscious processing.

## INTRODUCTION

‘*It is not the consciousness of men that determines their being, but, on the contrary, their social being that determines their consciousness.*’ Though perhaps conceived for a different purpose by Karl Marx (1904: 11), a neuroscientific interpretation of the notion of socially constructed consciousness has received both theoretical and empirical support in recent years (Frith, 2010; Graziano and Kastner, 2011). One powerful method for investigating how conscious experience is affected by socially relevant information is continuous flash suppression (CFS). An image of interest is presented to one eye while a sequence of rapidly flickering arrays of randomly generated ‘Mondrian’ masks is shown to the other eye. This configuration suppresses the image of interest from awareness for a considerable period (Tsuchiya and Koch, 2005). The time it takes for the suppressed image to break into awareness can be used as a probe for preconscious visual processing (Jiang *et al.*, 2007; Yang *et al.*, 2007; Stein *et al.*, 2011). CFS has enabled several clear demonstrations of the relevance of social engagement for preconscious vision. For example, if faces suppressed from awareness using CFS have eye gaze directed toward the observer, they break through suppression and reach awareness faster than faces with averted gaze (Stein *et al.*, 2011). Social traits inferred from the appearance of suppressed faces, such as dominance and trustworthiness (Oosterhof and Todorov, 2008), also impact on preconscious visual processing as measured using CFS (Stewart *et al.*, 2012). Similarly, faces displaying emotional expressions also modulate such preconscious processing: fearful faces suppressed by CFS gain faster access to awareness than neutral or happy faces (Yang *et al.*, 2007), while schematic angry faces emerge from CFS more slowly (Stein and Sterzer, 2012). In neural terms, invisible fearful faces activate the amygdala (Whalen *et al.*, 1998; Williams *et al.*, 2004), as well as the fusiform face area (FFA) and superior temporal sulcus (STS; Jiang and He, 2006).

Facial emotional expression is highly relevant to social interaction, but traits such as attractiveness, dominance and trustworthiness are separate and also socially relevant facial attributes. Evaluation of attractiveness and dominance are of evolutionary importance (Thornhill and Gangestad, 1999; Adams *et al.*, 2011); trustworthiness evaluation also predicts important social outcomes (Todorov *et al.*, 2005). There is behavioral evidence to support commonality of mechanisms for evaluating emotions and traits of faces (Engell *et al.*, 2010; Said *et al.*, 2011), and a modest literature localizing the neural processing of facial attractiveness (e.g. Winston *et al.*, 2007) and trustworthiness (e.g. Winston *et al.*, 2002; Todorov *et al.*, 2008), which does indeed show overlap with areas involved in processing facial emotion. On the other hand, both as sociological and psychological constructs, and on the basis of neuroimaging evidence, social traits appear to be distinct from emotional expressions. Unlike emotional expressions, facial traits are a non-dynamic, and arguably more transparent and less fakeable, source of social information. Principal components analysis of a large data set of unconstrained descriptions of real-life face images shows that social face evaluation can be represented using two orthogonal dimensions of dominance and trustworthiness (Oosterhof and Todorov, 2008). At the extremes of these dimensions, faces are also rated as showing emotion (e.g. untrustworthy faces are rated as angry). However, this is not the case for more mild variations in dominance and trustworthiness; such faces are reliably rated as emotionally neutral (Oosterhof and Todorov, 2008). While there has been some investigation of preconscious evaluation of facial emotions, preconscious evaluation of social face traits has only recently been explored (Stewart *et al.*, 2012), and the neuronal correlates of such preconscious evaluation remain unknown. The model of Oosterhof and Todorov (2008) provides a useful framework for such exploration.

An important feature of emotional or social modulation of awareness is the substantial degree of between-participant variability, in both behavioral and neuronal indices. Self-reported mood and personality measures explain some of this variability. For example, scores from trait and state anxiety questionnaires predict how often an individual perceives angry versus happy faces during binocular rivalry (Gray *et al.*, 2009), while BOLD activation in amygdala and STS when viewing fearful faces masked by CFS correlates with negative affectivity score (Vizueta *et al.*, 2012). Variability in social perception correlates with local variability in neuronal function, as measured with both functional magnetic resonance imaging (fMRI; e.g. Vrtička *et al.*, 2011) and electroencephalography (e.g. Jetha *et al.*, 2012). Here, we hypothesized that regional variation in brain *structure* might also predict such variability in social perception. While a growing number of studies in recent years have explored relationships between brain structure and behavior (reviewed by Kanai and Rees, 2011), none have yet focused on possible correlations between brain structure and preconscious social perception.

We used an individual differences approach to examine the relationship between brain structure and a behavioral index of preconscious social evaluation. Individual variability in perception of face traits of dominance and trustworthiness outside of awareness is strongly correlated with scores on self-report questionnaires that reflect inclination to submissive behavior and propensity to trust others (Stewart *et al.*, 2012). We now determined whether individual differences in evaluation of dominance and trustworthiness, varied orthogonally using the model of Oosterhof and Todorov (2008), were associated with local variations in gray matter (GM) volume measured using structural magnetic resonance imaging (MRI). We hypothesized that behavioral measures of preconscious dominance evaluation would be associated with GM volume in the amygdala and right insula (Whalen *et al.*, 2001; Dannlowski *et al.*, 2007; Chiao *et al.*, 2008). Further, we hypothesized that behavioral measures of preconscious trustworthiness evaluation would be associated with GM volume in the amygdala, right insula, fusiform gyrus and medial prefrontal cortex (mPFC; Winston *et al.*, 2002; Todorov *et al.*, 2008).

## MATERIALS AND METHODS

### Participants

Thirty-six participants (23 female; mean ± s.d. age = 23.2 ± 4.6 years; range = 18–35 years) took part in the study. All were right-handed, had normal or corrected-to-normal vision and did not report any history of neurological or other major illness. Participants gave written informed consent, and experiments were approved by the local research ethics committee.

### Stimuli and display apparatus

**Fig. 1:**
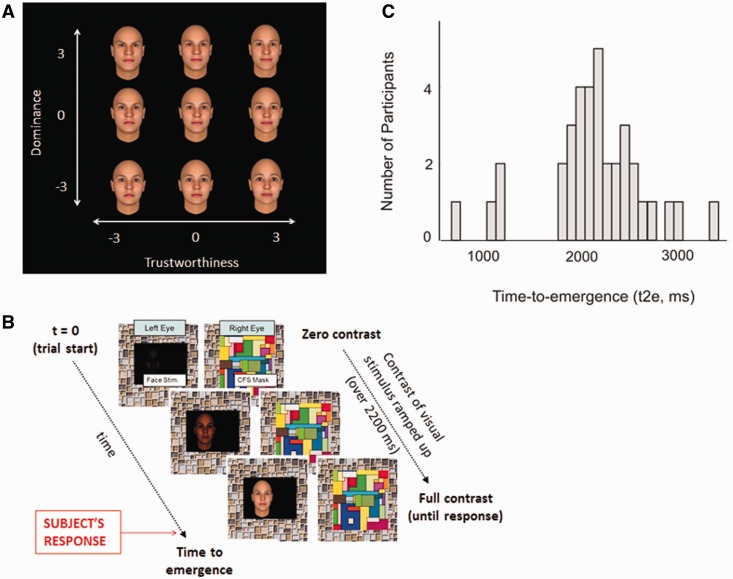
**(A)** Face stimuli. The two-dimensional trustworthiness-by-dominance space representing evaluation of social face traits (Oosterhof and Todorov, 2008). Trustworthiness and dominance vary in standard deviations from a neutral face along the x-axis and y-axis, respectively. The nine face images shown were all used in the behavioral paradigm. **(B)** Schematic representation of the behavioral paradigm. Images depict the temporal sequence of events that take place during each experimental trial. The CFS mask presented to one eye (the right eye in this case) changes with a frequency of 9.4 Hz. Subjects respond by pressing one of two buttons to indicate whether the face appears on the left or the right of the black box (in the image the correct response would be ‘left’). **(C)** Frequency distribution of t2e values. A histogram highlighting the substantial variability in mean t2e across individuals in our experimental sample.

We employed an identical set of stimuli and behavioral paradigm to those used in a recent study from our laboratory (Stewart *et al.*, 2012). A single randomly generated Caucasian male face image was produced using the Facegen Modeller programme, and parametrically manipulated along orthogonal axes of trustworthiness and dominance, using an extensively validated model (Oosterhof and Todorov, 2008). We used every permutation of dominance and trustworthiness, each at −3, 0 and +3 standard deviations from the neutral, resulting in nine versions of the same face identity (Figure 1A). At such degrees of variation in dominance and trustworthiness, faces are not mis-categorized as having any emotional expression (Oosterhof and Todorov, 2008).

The experimental paradigm was programmed using the Cogent Toolbox (http://www.vislab.ucl.ac.uk/cogent.php) for MATLAB (The Mathworks, Inc. Natick, MA). Stimuli were presented on a Sony Trinitron GDM-F520 monitor (1600 × 1200 at 85 Hz) and viewed through a mirror stereoscope mounted on a head and chin rest, with a black cardboard divider between the chin rest and screen. This ensured that each eye could see one side of the screen only and there was a stable base for fixation at a constant viewing distance of 65.5 cm. Two images were displayed side-by-side on the monitor, each with a central white fixation cross (0.6° visual angle) and tile frame surround (11.77° visual angle), upon a uniform gray background (background luminance = 65 Cd/m^2^). Optimal perceptual fusion of the two images was ensured before commencing each experiment. Responses were made with the right hand, using a computer keyboard pad.

### Behavioral procedures

A schematic version of the paradigm is shown in Figure 1B. For each trial, a dynamic and randomly generated colored noise pattern (changing at frequency 9.4 Hz) was presented to the non-dominant eye at full contrast, while the face image was presented on a black background to the dominant eye at a location 1 cm (0.7° visual angle) left or right of the fixation cross for that eye. The contrast of the face image was increased gradually from 0% to 100% during the initial 2200 ms of the trial and subsequently remained constant (Jiang *et al.*, 2007). Due to strong interocular suppression induced by the noise pattern, the face was rendered invisible to the participant for some time, before emerging from suppression and into awareness. Participants were instructed to make a button press (left or right arrow) as soon as they were confident that the face was visible either on the left or on the right side of central fixation. Both speed and accuracy were emphasized. Correct responses provided a measure of time-to-emergence (t2e) for the face (milliseconds from onset of stimulus presentation to button press). If no response had been made 10 s after the start of a trial, the trial terminated. Both incorrect-response and non-response trials were excluded from further analysis.

Participants completed 288 trials (eight blocks of 36 trials each) with each of the nine face versions presented 32 times (four times in each block). Before the beginning of the experiment, a 36-trial practice block was undertaken. Here, the eye presented with the face stimulus was randomized on each trial. For all participants, presenting the face to one eye resulted in a significantly shorter t2e (t2e values for two eyes were compared using a paired *t*-test). Following the procedure we developed previously (Stewart *et al.*, 2012) the eye resulting in shorter t2e was denoted the ‘dominant eye’ and subsequently all face images were presented to this eye.

### MRI data acquisition

High-resolution anatomical magnetic resonance (MR) images were obtained for all 36 participants on a separate occasion from behavioral testing using a 1.5-T Siemens Sonata MRI scanner (Siemens Medical, Erlangen, Germany). A T1-weighted 3-D Modified Driven Equilibrium Fourier Transform sequence (TR = 12.24 ms; TE = 3.56 ms; field of view = 256 mm × 256 mm; voxel size = 1 mm × 1 mm × 1 mm) was used.

### MRI data analysis

Voxel-based morphometric (VBM) analysis (Ashburner and Friston, 2000) was performed on the acquired imaging data. MR images were segmented for GM and white matter using the segmentation tools in SPM8 (http://www.fil.ion.ucl.ac.uk/spm). Subsequently, Diffeomorphic Anatomical Registration Through Exponentiated Lie Algebra (Ashburner, 2007) was performed in SPM8 for intersubject registration of the GM images. The registered images were smoothed with an 8 mm Gaussian kernel and then transformed to MNI stereotactic space using affine and non-linear spatial normalization implemented in SPM8 for multiple regression analysis.

Potentially confounding factors of gender identity and age, which affect brain structure (Good *et al.*, 2001; Smith *et al.*, 2007), were regressed out by modeling them as covariates of no interest. Global nuisance effects were accounted for by including the global covariate in the general linear model. Non-stationary cluster-level correction (Hayasaka *et al.*, 2004) was undertaken to improve the reliability of cluster-level statistics. We used *P* < 0.05 (family-wise error, FWE; corrected for whole brain volume) as the criterion for considering voxels as having a significant correlation with an individual’s behavioral measures.

The methods for several additional analyses, including additional VBM analyses as well as calculation of cortical thickness, surface area and volume, are described in Supplementary Methods.

## RESULTS

We presented face images, varying parametrically along orthogonal dimensions of dominance and trustworthiness, outside of awareness (under CFS). We recorded t2e, a measure that reflects the strength of each face image in competing for awareness. There was substantial interindividual variability of mean t2e across participants (mean t2e range: 0.63–3.39 s; Figure 1C). The bimodal appearance of the distribution of individual t2e in Figure 1C made us question whether the group of four participants with fastest t2e was exhibiting a different pattern of behavior compared with the rest of our sample. We therefore performed further analyses that allowed us to exclude any important differences between these groups. We found that the four fast-t2e individuals did not make more task errors, had similarly distributed response times and exhibited similar effects of facial dominance and trustworthiness when compared with other individuals in our experimental sample. Removing these four individuals from our behavioral and imaging analyses did not alter the pattern of experimental findings (see Supplementary Results for full details).

### Behavioral results: facial dominance and trustworthiness affect t2e

**Fig. 2:**
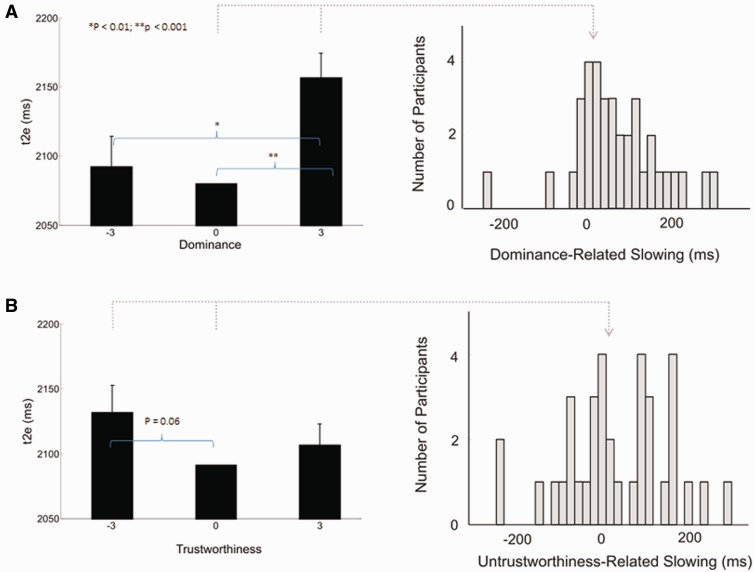
Effects of social face traits (**A**, dominance and **B**, trustworthiness) on t2e. In the left hand panels of the figure, mean values of t2e across all subjects (*n* = 36) are plotted along the *y* axis. Along the *x* axis are plotted dominance (A) and trustworthiness (B), in standard deviations from the neutral. For each level of dominance in A, t2e scores are collapsed across the three levels of trustworthiness, and for each level of trustworthiness in B, t2e scores are collapsed across the three levels of dominance. Error bars represent standard errors of mean difference between the specific condition and a neutral face. In the right hand panels of the figure, frequency distributions of individual values for (A) dominance-related slowing [t2e(+3dom) – t2e(neutral)], and (B) untrustworthiness-related slowing [t2e(−3trust) – t2e(neutral)] are shown. Substantial individual variability can be seen both for the measure derived from dominance and for that derived from trustworthiness.

Task error rates were low (mean error rate across participants = 2.2% of trials; see Supplementary Results for full details). We entered mean t2e scores for each of the nine face types into a two-way repeated-measures analysis of variance with factors of dominance and trustworthiness (three levels for each factor). This revealed a significant main effect of dominance [*F*_(2,70)_ = 8.88, *P* < 0.001], and a marginally significant main effect of trustworthiness [*F*_(2,70)_ = 2.45, *P* = 0.094]. Figure 2 depicts plots of the main effects of dominance and trustworthiness, with each collapsed across the other social trait dimension. There was no significant interaction between these two main effects [*F*_(4,140)_ = 1.34, *P* = 0.264]. Because we have used parametric statistical tests, we also log-transformed the behavioral data, confirming that this did not result in any changes to the results. Including gender identity and age in our statistical model did not change the pattern of results (see Supplementary Results for more details of these analyses). The results closely replicate previous findings in separate groups of participants, with some variation in statistical significance (in our previous results, main effects of both dominance and trustworthiness reached significance; Stewart *et al.*, 2012). Given the findings of Stewart *et al.*, as well as the findings presented here, we believe that the effect of face trustworthiness on t2e is highly variable across individuals, and thus, the presence of a significant group-level effect varies according to the experimental sample (see Supplementary Discussion for more details). *Post hoc* comparisons revealed that the main effect of dominance reflected significantly slower t2e for most-dominant faces relative to least-dominant faces [*t*_(35)_ = −3.41, *P* = 0.002], and to neutral-dominance faces [*t*_(35)_ = −4.31, *P* < 0.001]. The main effect of trustworthiness reflected borderline-significant slowing of t2e for least-trustworthy faces relative to neutral-trustworthiness faces [*t*_(35)_ = 1.98, *P* = 0.056]. Remaining pairwise comparisons were not significant (minimum *P* value = 0.166; see Supplementary Results for full details).

Based on the effects of facial dominance and trustworthiness, and the particularly clear differences in t2e between certain levels of these traits, a representative measure for individual strength of each of these dimensions of face evaluation was calculated as follows (see also Stewart *et al.*, 2012):

1. Dominance-related slowing (t2e_(+3dom)_ – t2e_(neutral)_)
2. Untrustworthiness-related slowing (t2e_(−3trust)_ – t2e_(neutral)_)

As with mean t2e, there was substantial interindividual variability in the size of the dominance-related slowing and untrustworthiness-related slowing effects (Figure 2). Dominance-related slowing and untrustworthiness-related slowing were not correlated (*r* = −0.10. *P* = 0.56).

### Brain structural correlates of unconscious evaluation of facial dominance/untrustworthiness

Next we tested our hypotheses that individual variability in dominance-related slowing and untrustworthiness-related slowing would be correlated with individual differences in local brain structure. Behavioral scores relating to both types of face trait were entered into the same SPM design matrix.

**Fig. 3:**
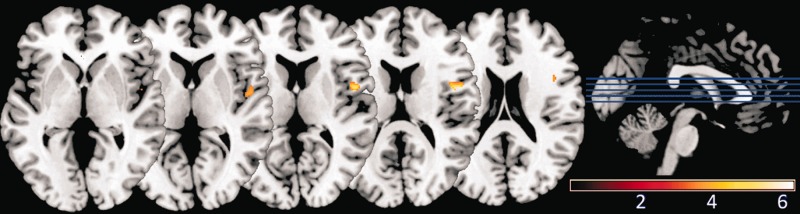
Structural brain correlates of individual differences in preconscious dominance-related slowing. A brain locus in right frontal operculum, where GM volume correlates significantly with behavioral effects of dominance-related slowing, is shown in color (from brown, representing low correlation, to white, representing high correlation), overlaid on a standard template brain. A threshold of *P* < 0.001 uncorrected has been used for display purposes. We used *P* < 0.05 (FWE corrected for whole-brain volume, or corrected for small volume around coordinates predicted a priori) as the threshold below which to consider voxels as having a significant correlation with an individual’s behavioral measures. Colorbar scale represents *T* values.

**Fig. 4:**
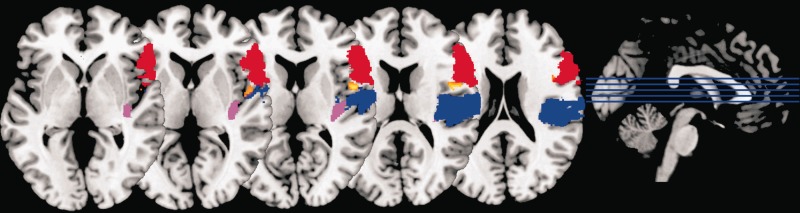
Regions near the locus for dominance-related slowing, defined according to the probabilistic cytoarchitectonic maps of the SPM Anatomy Toolbox. The locus in right frontal operculum (as described in Figure 3) is shown in color (from brown, representing low correlation, to white, representing high correlation), along with anatomical masks derived from the SPM Anatomy toolbox for posterior insula (violet), IFG (red) and SII (blue). The VBM result and three masks are overlaid on a standard template brain. The location of our result in between areas defined as posterior insula, IFG and SII is apparent.

**Fig. 5:**
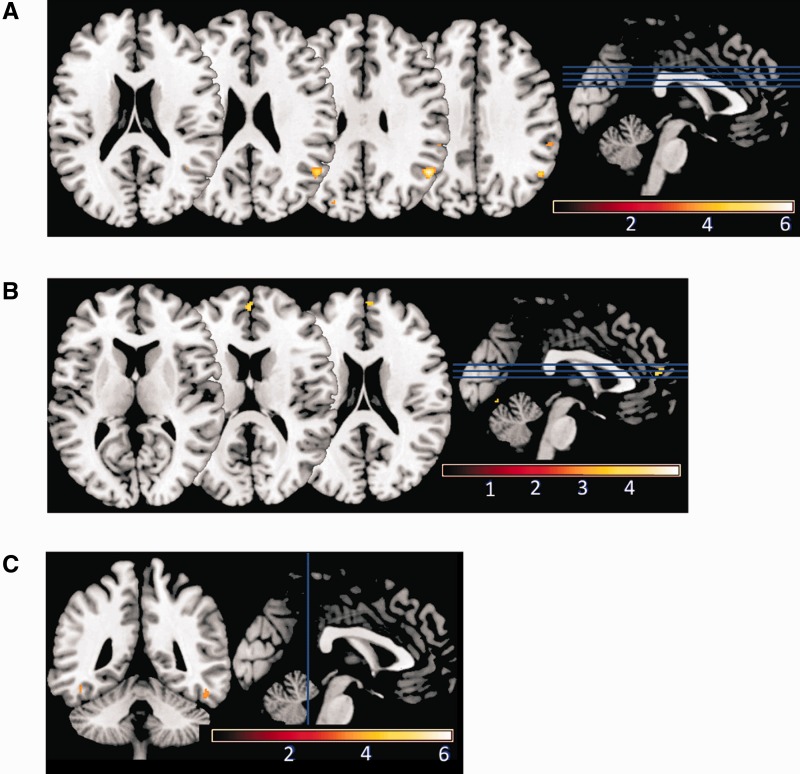
Structural brain correlates of individual differences in preconscious untrustworthiness-related slowing. Brain loci in (**A**) pTPJ; (**B**) mPFC; and (**C**) bilateral fusiform gyrus, where GM volume correlates significantly with behavioral effects of untrustworthiness-related slowing are shown in color (from brown, representing low correlation, to white, representing high correlation), overlaid on a standard template brain. A threshold of *P* < 0.001 uncorrected has been used for display purposes. We used *P* < 0.05 (FWE corrected for whole-brain volume, or corrected for small volume around coordinates predicted a priori) as the threshold below which to consider voxels as having a significant correlation with an individual’s behavioral measures. Color bar scales represent *T* values.

GM volume in right frontal operculum was significantly correlated with individual differences in dominance-related slowing (*x* = 48, *y* = 2, *z* = 13; *T* = 6.27; *Z* = 4.97; *P*_FWE-corr_ = 0.016; Figure 3; Table 1). The Anatomy Toolbox for SPM (http://www.fz-juelich.de/inm/inm-1/DE/Forschung/_docs/SPMAnatomyToolbox/SPMAnatomyToolbox_node.html) indicates that this region is located between insula, inferior frontal gyrus (IFG) and secondary somatosensory cortex (SII; Figure 4). Using the probabilistic cytoarchitectonic maps provided by Anatomy Toolbox, we found that there was a 10% probability that this locus was located in IFG (Brodmann 44) and a 10% probability that it was located in SII. Meanwhile, GM volume in right posterior temporoparietal junction (pTPJ) was significantly correlated with individual differences in untrustworthiness-related slowing (*x* = 51, *y* = −57, *z* = 31; *T* = 6.14; *Z* = 4.90; *P*_FWE-corr_ = 0.022; Figure 5A; Table 1). Both findings were statistically significant after FWE correction for whole-brain volume. In addition, both correlations were negative, indicating that *reduced* GM volume in right frontal operculum and right pTPJ predicts *increased* dominance-related slowing and untrustworthiness-related slowing, respectively.

**Table 1:** VBM analysis: whole-brain statistics

Behavioral effect	Corr	Location		MNI coordinates			Statistics		
		Region	Hem	*x*	*y*	*z*	*T*	*Z*	*P*_FWE-corr_
Dominance-related slowing	Neg	Frontal operculum	Right	48	2	13	6.27	4.97	0.016
Untrustworthiness-related slowing	Neg	TPJ	Right	51	−57	31	6.14	4.90	0.022

Coordinates and statistical results for peak voxels where GM volume was significantly correlated with dominance-related slowing or untrustworthiness-related slowing (*P* < 0.05, FWE corrected for multiple comparisons across whole-brain volume). Corr, direction of correlation; Hem, hemisphere; *P*_FWE-corr_, family-wise error-corrected *P*-value; TPJ, temporoparietal junction.

**Fig. 6:**
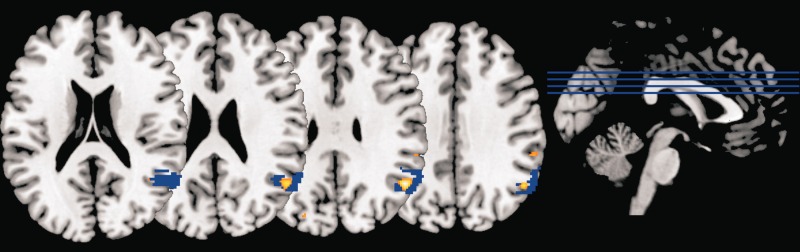
Correspondence between right pTPJ result of present study and previously described TPJp subregion. The TPJp subregion described by Mars *et al.* (2011) is shown in blue and the right pTPJ cluster for which we have shown a significant correlation with untrustworthiness avoidance (Table 1; Figure 5A) is shown in color (from brown, representing low correlation with untrustworthiness-related slowing, to white, representing high correlation). The loci are overlaid on a standard template brain. A substantial degree of overlap is seen between our pTPJ finding and the TPJp subregion, known to have strong resting-statefunctional connectivity with other brain regions involved in social cognition (Mars *et al.*, 2011).

Right TPJ has been divided into three subregions based on diffusion-weighted tractography and resting state functional connectivity (Mars *et al.*, 2011). We used mask images for each of these subregions to determine within which of them our reported right pTPJ cluster falls. Small-volume correction using the most posterior TPJ mask (TPJp) resulted in a similar result to our whole-brain pTPJ finding (*x* = 51, *y* = −58, *z* = 31; *T* = 5.77; *Z* = 4.70; *P*_FWE-corr_ < 0.001), confirming that the majority of our TPJ cluster falls within the TPJp region described by Mars and colleagues (Figure 6).

We had predicted *a priori*, based on existing evidence, correlations between our behavioral measures and GM volume in a number of brain regions (Table 2). To test these predictions, we employed small volume correction for multiple comparisons within a sphere with 15 mm radius (15 mm arbitrarily chosen for brain regions of unknown or highly variable size, as previously employed by Kanai *et al.*, 2010) centered at each of the coordinates listed in Table 2. For fusiform gyrus and amygdala, smaller spheres with 8 mm radius were used for small-volume correction, since the volume of such a sphere (2550 voxels of 1 mm^3^) roughly matches the volume of functionally relevant portions of these regions according to meta-analyses (Joseph, 2001; Costafreda *et al.*, 2008). A threshold of *P* < 0.05 (FWE-corrected for small volume) was used as the criterion for significance. There was a significant positive correlation between mPFC GM volume and untrustworthiness-related slowing (*x* = −2, *y* = 54, *z* = 13; *T* = 4.15; *Z* = 3.66; *P*_FWE-corr_ = 0.027; Figure 5B; Table 3). This region shows a non-linear BOLD response to changes in face trustworthiness (Todorov *et al.*, 2008; *x* = 2, *y* = 65, *z* = 10). GM volume in fusiform gyrus bilaterally was correlated significantly and negatively with untrustworthiness-related slowing (*x* = −47, *y* = −45, *z* = −18; *T* = 3.52; *Z* = 3.19; *P*_FWE-corr_ = 0.023 for left fusiform; and *x* = 50, *y* = −44, *z* = −20; *T* = 3.67; *Z* = 3.31; *P*_FWE-corr_ = 0.017 for right fusiform; Figure 5C; Table 3). Bilateral fusiform gyrus is differentially activated by faces that vary in trustworthiness (Winston *et al.*, 2002; *x* = −48, *y* = −48, *z* = −24; and *x* = 44, *y* = −46, *z* = −22). GM volume in right frontal operculum was negatively correlated with dominance-related slowing (*x* = 48, *y* = 2, *z* = 13; *T* = 6.27, *P*_FWE-corr_ < 0.001). In this case, small volume correction was centered at nearby right insula, which has previously been activated by untrustworthy faces (Winston *et al.*, 2002; *x* = 42, *y* = −4, *z* = 12) and dominant head postures (Chiao *et al.*, 2008; *x* = 39, *y* = 9, *z* = 15). This result refers to the same locus in right frontal operculum reported for whole-brain analysis (Table 1) and is therefore not discussed further. However, it demonstrates that the location of our right frontal opercular locus is not distant from coordinates previously reported in right insula with respect to processing of dominance and/or anger (Whalen *et al.*, 2001; Dannlowski *et al.*, 2007; Chiao *et al.*, 2008). We examined the scatterplots relating to all of the VBM results described to ensure they were driven by linear relationships rather than outliers (see Supplementary Results).

**Table 2:** Predicted regions of interest for small volume correction analysis

Location		MNI coordinates			Source	Significant result
Region	Hem	*x*	*y*	*z*		
Amygdala	L	−24	−2	−22	Harvard-Oxford Subcortical Structural Atlas	No
Amygdala	R	26	0	−22	Harvard-Oxford Subcortical Structural Atlas	No
Putamen	L	−16	13	−4	Todorov *et al.*, 2008	No
Precuneus		−1	−61	39	Todorov *et al.*, 2008	No
mPFC		1	59	24	Todorov *et al.*, 2008	Yes^a^
Fusiform G	L	−48	−48	−24	Winston *et al.*, 2002	Yes^b^
Fusiform G	R	44	−46	−22	Winston *et al.*, 2002	Yes^b^
pSTS	R	56	−44	4	Winston *et al.*, 2002	No
Insula	R	42	−4	12	Winston *et al.*, 2002	Yes^c^
Lingual Gyrus	R	27	−54	13	Chiao *et al.*, 2008	No
Sup. Temp. G	R	60	−51	3	Chiao *et al.*, 2008	No
Insula	R	39	9	15	Chiao *et al.*, 2008	Yes^c^

Coordinates used for the small volume correction analysis (as described in Materials and Methods section). Some of the spheres centered at these coordinates contained voxels in which GM volume was significantly correlated with either dominance-related slowing or untrustworthiness-related slowing (as indicated in final column of table below). Hem, hemisphere; mPFC, medial prefrontal cortex; Fusiform G, fusiform gyrus; pSTS, posterior superior temporal sulcus; Sup. Temp G, superior temporal gyrus.

^a^Positive correlation between untrustworthiness-related slowing and GM volume in mPFC; see Table 3 for details.

^b^Negative correlation between untrustworthiness-related slowing and GM volume in bilateral fusiform gyrus; see Table 3 for details.

^c^Negative correlation between dominance-related slowing and GM volume in the same locus as reported in whole-brain analysis (see Table 1).

**Table 3:** VBM analysis: small-volume statistics

Behavioral effect	Corr	Location		MNI coordinates			Statistics		
		Region	Hem	*x*	*y*	*z*	*T*	*Z*	*P*_FWE-corr_
Untrustworthiness avoidance	Pos	mPFC (see Table 2)		−2	54	13	4.15	3.66	0.027
Untrustworthiness avoidance	Neg	Fusiform G (see Table 2)	L	−47	−45	−18	3.52	3.19	0.023
Untrustworthiness avoidance	Neg	Fusiform G (see Table 2)	R	50	−44	−20	3.67	3.31	0.017

Coordinates and statistical results for peak voxels, within 15 mm or 8 mm spheres used for small-volume correction, where GM volume was significantly correlated with dominance-related slowing or untrustworthiness-related slowing (*P* < 0.05, FWE corrected for multiple comparisons across a 15 mm or 8 mm sphere centered at coordinates specified in Table 2). Corr, direction of correlation; Hem, hemisphere; *P*_FWE-corr_, family-wise error-corrected *P*-value; mPFC, medial prefrontal cortex; Fusiform G, fusiform gyrus.

There were no correlations between GM volume and either dominance-related slowing or untrustworthiness-related slowing after small-volume correction in left or right amygdala. Given this surprising null result, we also performed separate analyses to determine the correlation between untrustworthiness-related slowing or dominance-related slowing and amygdala volume calculated using automated subcortical segmentation (Supplementary Methods and Supplementary Results). However, we found no correlation between the volume of the amygdala and either of our behavioral measures.

The structural correlates of individual differences in dominance-related slowing and untrustworthiness-related slowing appear at least partially dissociable (see Supplementary Results for details of further analyses to support this claim). We also performed further VBM analyses to explore any correlation between local brain structure and task error rate as well as t2e for faces across all levels of dominance and trustworthiness (see Supplementary Material).

## DISCUSSION

We explored whether brain structure was correlated with individual differences in preconscious evaluation of facial dominance and trustworthiness. Based on previous experimental findings we predicted overlap in the structural correlates of preconscious processing for these two socially relevant facial traits. Instead, we found that these neural correlates were dissociable: preconscious slowing of dominance evaluation was negatively correlated with GM volume in right frontal operculum, while preconscious slowing of untrustworthiness evaluation was negatively correlated with GM volume in right pTPJ and bilateral fusiform gyrus, and positively correlated with GM volume in mPFC. This dissociation suggests that preconscious evaluation of dominance and untrustworthiness is linked to at least partially separable neural substrates.

### Dominance-related slowing

We found that reduced GM volume in the right frontal operculum was correlated with increased slowing of preconscious processing of dominant faces. Previous fMRI studies have reported activation in a nearby region in the middle portion of right insula during viewing of dominant head postures (Chiao *et al.*, 2008), or angry faces (Dannlowski *et al.*, 2007). Taken together, these results suggest that adjacent regions are involved in dominance evaluation both when it depends on relatively invariant face traits, and when it depends on more view-specific and dynamic cues such as head posture. There is evidence to suggest that insula and frontal operculum may have common or at least closely related functional roles, for example, in representing taste (O’Doherty *et al.*, 2001), facilitating empathy for others’ emotions (Jabbi *et al.*, 2007) and interpreting social intentions (Gobbini *et al.*, 2007).

The human insula is involved in the neural processing of emotion (Phan *et al.*, 2002). A number of fMRI studies have also demonstrated insula activation associated with risky decisions (e.g. Paulus *et al.*, 2003; Clark *et al.*, 2008). Paulus *et al.* (2003) focused on individual differences and reported that insula activation was correlated with self-measures of harm avoidance and neuroticism. These studies point to a nuanced role for the insula in evaluating risks and possibly balancing approach versus avoidance of risky situations and/or conspecifics.

The neural mechanisms for evaluation of social face traits likely overlap those for evaluation of emotional facial expressions. One proposed mechanism for emotion recognition is the engagement of mirror systems, which enable simulation of the observed emotion in the perceiver. Both quantitative lesion mapping (Adolphs *et al.*, 2000), and suppression of activity using repetitive transcranial magnetic stimulation (Pourtois *et al.*, 2004; Pitcher *et al.*, 2008) show that right somatosensory cortex is causally important for facial emotion recognition. Our locus in frontal operculum is very close to secondary somatosensory area SII. Whether this close proximity plays a role in bringing social and emotional evaluation of faces together remains to be tested. Alternatively, our result may relate more closely to the role of IFG in face perception, which also has links to the proposed emotional mirror systems (Jabbi *et al.*, 2007; Shamay-Tsoory *et al.*, 2009).

The negative correlation between GM volume in frontal operculum and dominance-related slowing may be understood in terms of availability of processing resources for preconscious information. A previous meta-analysis showed that the insula is consistently activated by conditioned threat (Pessoa, 2009). Furthermore, such increased activation was associated with impaired behavioral performance, for example, reduced accuracy in a letter detection task. Pessoa (2009) hypothesized that such impairment in behavioral performance in the face of increased neural activation may reflect recruitment of attentional and effortful control, engaging ‘common-pool’ executive resources also needed for inhibition, shifting and updating, which are all necessary for successful behavioral performance. Socially threatening stimuli automatically engage attention, and such engagement is dependent on availability of processing resources (Huang *et al.*, 2011). We suggest that in our experimental paradigm, the threat conveyed by dominant faces may similarly result in engagement of processing resources in right frontal operculum (instead of insula as above), limiting resources available for risk evaluation or social appraisal. Such limitation would potentially be more severe for individuals who have relatively less GM in this opercular region, manifesting as increased slowing of preconscious dominance evaluation.

### Untrustworthiness-related slowing

Individual differences in slowing of preconscious processing for untrustworthy faces were correlated with local GM volume in a distributed group of brain regions. Reduced GM volume in right pTPJ and bilateral fusiform gyrus and increased GM volume in mPFC all predicted increased untrustworthiness-related slowing. These findings only partially confirmed our predictions that untrustworthiness-related slowing would co-vary with GM volume in the amygdala, insula, fusiform gyrus and mPFC (Winston *et al.*, 2002; Todorov *et al.*, 2008).

Our findings in bilateral fusiform gyrus are consistent with this region’s responsiveness to social cues (Fox *et al.*, 2009), including facial trustworthiness in particular (Winston *et al.*, 2002). However, further investigation will be required to understand whether the functional role of fusiform gyrus in trustworthiness evaluation relates to differences in physical appearance and configuration of features, or to evaluation more directly of socially relevant attributes, such as emotional content, or inference regarding goals and intentions.

GM volume in mPFC was positively correlated with preconscious untrustworthiness-related slowing. Substantial converging evidence implicates mPFC in tasks that depend on mentalizing or ‘theory of mind’ (the making of sophisticated inferences about the goals and intentions of others; Gallagher and Frith, 2003; Saxe and Kanwisher, 2003; Amodio and Frith, 2006; Van Overwalle, 2009). The expanding fMRI literature on mPFC function has led to proposed functional subdivisions of this region, with one framework suggesting subdivisions into a posterior rostral region (involved in cognitive tasks such as action monitoring), an anterior rostral region (involved in emotional and social tasks) and an orbital region (linked with monitoring of punishment and reward; Amodio and Frith, 2006). The structural locus identified in our results (Table 3) lies within the anterior rostral subregion, which is involved in a wide variety of social cognitive tasks (Mitchell *et al.*, 2005; Amodio and Frith, 2006).

An unexpected but statistically robust finding was the negative correlation between GM volume in pTPJ and untrustworthiness-related slowing. This region is widely implicated in processes of social cognition (Saxe and Kanwisher, 2003; Van Overwalle, 2009) and as such is consistent with our finding in mPFC. While TPJ is involved both in theory of mind and reorienting of attention (Decety and Lamm, 2007), our result is in the posterior portion of TPJ, which has strong resting-state functional connectivity with a number of regions implicated in social cognition, including mPFC, posterior cingulate and precuneus (Mars *et al.*, 2011). Previous studies have implicated pTPJ in social perception and mentalizing, particularly assessment of similarity between self and the faces of others (Mitchell *et al.*, 2005), consequential decision making (Turk *et al.*, 2004) and reasoning about another person’s mental state (Saxe and Kanwisher, 2003). Our findings now extend the role of right pTPJ to include individual differences in preconscious social evaluation of faces based on untrustworthiness.

The negative correlation between GM volume in fusiform gyrus and untrustworthiness-related slowing may be interpreted in a similar fashion to our earlier discussion of dominance-related slowing and frontal operculum. fMRI studies show increased activation in FFA in response to threatening, as compared with neutral, faces (Anderson *et al.*, 2003). Moreover, individual differences in engagement of attention by threatening faces are tracked by neural activity in FFA, among other regions (Reeck *et al.*, 2012). Specifically, activation is stronger in individuals who exhibit delayed responses in the context of social threat. It may be that such increased activation is indicative of engagement of limited processing resources, which are then less available for other perceptual and evaluative functions performed by the fusiform gyrus. While regions of parietal cortex are involved in deployment of attention in relation to threatening stimuli (Pourtois and Vuilleumier, 2006), and TPJ also shows increased activation to threatening faces (Kret *et al.*, 2011), we are not aware of any evidence that enhanced activation in TPJ can be seen in association with slowed behavioral performance in the context of social threat, and this would be an intriguing possibility to explore in the future. Such a finding would support a similar interpretation for our findings in TPJ to the one offered for our findings in frontal operculum and fusiform gyrus.

The positive correlation between untrustworthiness-related slowing and GM volume in mPFC could be interpreted by considering the proposed role of mPFC in exerting inhibitory control over other regions involved in social evaluation. An inverse relationship between mPFC and amygdala activation during emotional appraisal and regulation is well described (Etkin *et al.*, 2011); moreover, microelectrode stimulation of mPFC in animals results in reduced responsiveness of the central nucleus of the amygdala (Quirk *et al.*, 2003). Top-down inhibitory relationships between mPFC and other regions involved in social evaluation such as TPJ (FeldmanHall *et al.*, 2013) and insula (Thom *et al.*, 2012) are also beginning to emerge. In this context, the opposite correlations between untrustworthiness-related slowing and GM volume in mPFC (as compared with the correlations with GM in TPJ and fusiform gyrus) could be interpreted to reflect mPFC’s inhibitory influences on earlier levels of hierarchical processing.

We note that GM volume, as measured by VBM, is a mixed measure, which subsumes both cortical surface area and cortical thickness (Hutton *et al.*, 2009). While variation in cortical surface area may imply differences in availability of processing power, variation in cortical thickness may imply differing laminar microstructure and connectivity. Determining which of these measures contributes more to our findings would have important implications for the interpretation of our results. We therefore performed additional cortical surface-based analyses to explore whether individual differences in our behavioral measures were correlated with cortical thickness or cortical surface area in any focal brain regions. We found no significant whole-brain corrected correlations between behavioral indices and cortical thickness or surface area anywhere in the brain (further details in Supplementary Methods and Supplementary Results). Our failure to extend our VBM findings by demonstrating similar relationships between cortical thickness or surface area and behavior may be the result of differences between the analysis methods used to derive these different structural measures. Volumetric measures of the same brain images made in Freesurfer and in SPM can differ by as much as 20% (Klauschen *et al.* 2009). We hope that future refinement in measurement methods for cortical structural indices will enable a clearer answer regarding which elements of brain structure relate to the demonstrated individual differences in perceptual processing.

Previous relevant findings, on which both our experimental predictions and the interpretation of our results are based, relate to *functional,* rather than structural, neuronal correlates of social perception. It is important to point out here that because relationships between brain structure and function are not fully clear, the direct comparisons between structural and functional brain correlates of behavior made throughout this manuscript rest on a number of assumptions that will need to be directly evaluated in the future.

Our failure to find a correlation between dominance or untrustworthiness-related slowing and amygdala structure (either GM volume or overall volume as derived from subcortical segmentation) is surprising given the wealth of evidence linking the amygdala to processing of trustworthiness and anger, both from fMRI (Whalen *et al.*, 2001; Winston *et al.*, 2002; Dannlowski *et al.*, 2007; Todorov *et al.*, 2008), and from lesion studies (Calder, 1996; Adolphs *et al.*, 1998). In addition, there is robust activation in amygdala to non-conscious emotionally relevant stimuli (Whalen *et al.*, 1998; Williams *et al.*, 2004). It is difficult to offer an explanation of this null finding, other than to emphasize that the lack of correlation between individual differences in our behavioral measures and structural measures in amygdala in no way excludes an important role for the amygdala in preconscious evaluation of face traits. This region may have a central role in such processes without having a significant relationship (at least as far as its GM volume is concerned) with individual differences in the associated behavioral phenomena. The use of functional imaging modalities will be an important next step in exploring more fully the proposed role of the amygdala in preconscious social face evaluation.

## CONCLUSIONS

Our results demonstrate that individual differences in preconscious social evaluation are associated with variability in local brain structure. Both dominant and untrustworthy faces may be processed as threatening stimuli and activate subcortical emotional and threat-response mechanisms. However, our findings that GM volume in distinct cortical regions was correlated with individual effects of preconscious dominance-related slowing (frontal operculum) and untrustworthiness-related slowing (pTPJ, mPFC and fusiform gyrus) support the notion that evaluation of these traits depends on at least partially separable neural substrates. Furthermore, our results show that even when performed outside of awareness, social evaluation relates to GM volume in regions subserving high-level processes of social cognition.

## SUPPLEMENTARY DATA

Supplementary data are available at *SCAN* online.

Supplementary Data
