# Supplementary material for: Human brain structure predicts individual differences in preconscious evaluation of facial dominance and trustworthiness
Source: Soc Cogn Affect Neurosci. 2014 Sep 4;10(5):690–9. doi: 10.1093/scan/nsu103 (PMC4420744; doi:10.1093/scan/nsu103)
Supplement: Supplementary Data [file supp_10_5_690__index.html]

Human brain structure predicts individual differences in preconscious evaluation of facial dominance and trustworthiness — Supplementary Data 

# Human brain structure predicts individual differences in preconscious evaluation of facial dominance and trustworthiness

## Supplementary Data

file

**Files in this Data Supplement:**

- Supplementary Data - doc file
