# Supplementary material for: Human brain structure predicts individual differences in preconscious evaluation of facial dominance and trustworthiness
Source: Soc Cogn Affect Neurosci. 2014 Sep 4;10(5):690–9. doi: 10.1093/scan/nsu103 (PMC4420744; doi:10.1093/scan/nsu103)
Supplement: Supplementary Data [file supp_nsu103_Getov_et_al_SupplementaryMaterial.doc]

**Supplementary Material**

Supplementary Methods

**Additional VBM analysis: Brain structural correlates of time-to-emergence and response errors**

In addition to the VBM analysis to assess correlations between local GM volume and behavioral measures of dominance and trustworthiness evaluation, we also performed two further separate analyses to determine (1) whether GM volume in any focal brain region was correlated with individual differences in mean t2e (collapsed across all levels of dominance and trustworthiness), and (2) whether GM volume in any focal brain region was correlated with individual error rate (% of experimental trials where an incorrect response was given). As in our main VBM analysis, described in the Methods section, we regressed out potentially confounding factors of gender identity and age, included the global covariate in the general linear model, and performed non-stationary cluster-level correction. As previously, we used p<0.05 (FWE-corrected for whole brain volume) as the criterion for considering voxels as having a significant correlation with an individual’s behavioral measures. We also performed small-volume correction analyses using the same methodology and regions of interest (including bilateral amygdala and bilateral fusiform gyrus), as those described in the Methods section.

**Amygdala volume calculation using subcortical structure segmentation**

To provide a structural measure reflecting amygdala volume that was independent from our VBM analysis, we computed the volume of both left and right amygdala from the MR image of each participant using automated subcortical structure segmentation (Fischl et al., 2002) implemented in Freesurfer Stable v5.3.0 ([http://surfer.nmr.mgh.harvard.edu](http://surfer.nmr.mgh.harvard.edu/)). These data were used for further analysis as described in Supplementary Results.

**Correlation between behavioral measures and cortical thickness, surface area and volume**

Gray matter volume, as measured by VBM, provides a mixed measure of cortical gray matter, which subsumes both cortical surface area and cortical thickness (Hutton et al., 2009). We therefore performed further analyses to compute cortical thickness, cortical surface area and volume using surface-based methods implemented in Freesurfer.

Reconstruction of the pial surface and GM/WM boundary was performed using each participant’s T1-weighted MR image, according to a fully automated procedure (Fischl et al., 1999). Once a complete cortical model had been generated, deformable procedures could be performed, including creation of surface based data such as maps of curvature and sulcal depth. This enabled computation of cortical thickness, surface area, and volume. Cortical thickness was computed as the shortest distance between the GM/WM boundary and the pial surface (Fischl and Dale, 2000). The pial surface and thickness data were transformed to a standard brain (fsaverage) using a surface-based coregistration implemented in Freesurfer. The thickness and area data were smoothed with a Gaussian kernel (full-width/half-max 10mm). For each of the measures of cortical thickness, surface area, and volume, a separate multiple regression model was constructed to identify cortical regions where the structural measure under examination was correlated with either of our behavioral measures (dominance-related slowing and untrustworthiness-related slowing). As for our VBM analyses, both gender identity and age of participants, as well as the behavioral measure not being evaluated, were included in the multiple regression analysis design matrix as covariates of no interest. In this way, we were able to regress out any effects attributable to these variables. Cluster-wise correction for multiple comparisons was performed using Monte Carlo simulation (Hagler et al., 2006). We used a cluster-wise threshold of p<0.05 as the criterion for significance.

Supplementary Results

**Additional behavioral results**

There were no significant differences between t2e for neutral-dominance and least-dominant faces [t_(35)_ = 0.56, *P* = 0.580], between t2e for neutral-trustworthiness and most-trustworthy faces [t_(35)_ = -0.04, *P* = 0.355], or between t2e for least-trustworthy and most-trustworthy faces [t_(35)_ = 1.42, *P* = 0.166].

Rates of incorrect responses in the left/right behavioral task (expressed as percentage of all responses) were low (mean 2.2%, range 0 – 7.6%, with four of the 36 participants making no errors at all), suggesting that all participants understood the task and performed it with care. There were no significant correlations between error rates and t2e [r = 0.04, p = 0.82], error rates and dominance-related slowing [r = -0.23, p = 0.18], or error rates and untrustworthiness-related slowing [r = 0.03, p = 0.89].

Log transforming the behavioral data resulted in very minimal changes to the statistical results: main effect of dominance [F_(2,70)_ = 9.04, P<0.001]; main effect of trustworthiness [F_(2,70)_ = 2.54, P = 0.086]; interaction [F_(4,140)_ = 1.29, P = 0.282]. *Post hoc* comparisons again showed significant differences between most-dominant and least-dominant faces [t_(35)_ = -3.64, P = 0.001] as well as most-dominant and neutral-dominance faces [t_(35)_ = -4.48, P < 0.001], and a borderline-significant difference between least-trustworthy and neutral-trustworthiness faces [t_(35)_ = 2.02, P = 0.051]. Given the high similarity of these results to the untransformed data, we used the untransformed data in our analyses.

We sought to check whether inclusion of gender identity and age into the statistical analysis of our behavioral data would have an influence on the findings. A repeated measures ANOVA with within-subject factors of dominance and trustworthiness (3 levels for each factor), and between-subject factors of gender identity and age, revealed a significant main effect of dominance [F_(2,34)_ = 4.75, P=0.015], and a non-significant main effect of trustworthiness [F_(2,34)_ = 1.57, P = 0.223]. The main effects of gender identity [F_(1,17)_ = 0.80, P=0.385] and age [F_(13,17)_ = 0.53, P=0.878] were not significant. There was a borderline-significant dominance*gender identity*age interaction [F_(8,34)_ = 2.20, P = 0.053]. None of the other interaction terms reached statistical significance. Despite the dominance*gender identity*age interaction, the pattern of our main effect of dominance is not altered from that described in the main manuscript. The pattern of the remaining results is also not changed by including gender identity and age in the analysis.

**Testing for the presence of outliers in our behavioral results**

Given the bimodal appearance of the distribution of mean individual t2e results in Figure 1C, we sought to determine whether the four individuals with fast t2e (<1.5 seconds) were in any way different to the remainder of the experimental sample. Using Chauvenet’s criterion, only the individual with fastest mean t2e (0.63 seconds) was classed statistically as an outlier. The mean error rate of the four participants in question was 1.3% (range 0.35 – 2.08%), which is even lower than the mean error rate for the whole experimental sample. We thus have no evidence that these participants were performing the experimental task incorrectly. Response times for each of these four participants approximated well to gamma distributions (Figure S1, top row), as do those for a representative selection of the remaining subjects (Figure S1, bottom row).

Examining the effect of dominance and trustworthiness on t2e at the individual subject level for each of the four fast-t2e participants showed that for two of them the trend for the main effects of dominance and trustworthiness closely resembles the group-level findings (plots S9 and S33 in Figure S2). For the other two participants (plots S12 and S27 in Figure S2), these trends are only partially in keeping with the group-level findings. However, there are a number of other participants (who do not have unusually fast t2e) for whom this is also the case (e.g. dominance plot for S31 and trustworthiness plot for S13 in Figure S2). We therefore have no strong indication that the individual effects of dominance and trustworthiness on t2e are unusual in the fast-t2e individuals.

The four fast-t2e individuals are not found at the extreme ends of the distributions of dominance-related slowing and untrustworthiness-related slowing scores (which are the behavioral indices used in the VBM analysis; Figure S3; the time bins in which fast-t2e participants fall are colored in red).

Removing the four individuals with fast t2e (<1.5 seconds) from our analyses did not alter the pattern of our findings, although the statistical significance was partially reduced, likely due to reduction in sample size. There remained a significant main effect of dominance [F_(2,62)_ = 8.13, P = 0.001]; the main effect of trustworthiness was reduced in strength [F_(2,62)_ = 1.55, P = 0.221], and as previously there was no significant interaction [F_(4,124)_ = 1.10, P = 0.358]. *Post hoc* comparisons revealed significant differences between most-dominant and least-dominant faces [t_(31)_ = -3.07, P = 0.004] and between most-dominant and neutral-dominance faces [t_(31)_ = -4.23, P < 0.001], and a persisting but weakened trend towards a difference between least-trustworthy and neutral-trustworthiness faces [t_(31)_ = 1.60, P = 0.119]. In terms of imaging results, the correlation between dominance-related slowing and GM volume in right frontal operculum remained present (locus at the same coordinates, though no longer significant at the whole-brain level: x = 48, y = 2, z = 13; T = 5.54; Z = 4.46; *P*_FWE-corr_ = 0.110) when these four participants were excluded from analysis. Likewise, the correlations between untrustworthiness-related slowing and GM volume in right pTPJ, bilateral fusiform gyrus and mPFC remained present after removal of these four participants, although with some minor changes in statistical significance. The result in pTPJ became only borderline-statistically significant at the whole-brain level (x = 51, y = -57, z = 30; T = 5.88; Z = 4.65; *P*_FWE-corr_ = 0.055); the small-volume-corrected results in left and right fusiform gyrus remained statistically significant (x = -47, y = -43, z = -18; T = 3.34; Z = 3.02; *P*_FWE-corr_ = 0.032 for left fusiform; and x = 50, y = -42, z = -21; T = 4.20; Z = 3.64; *P*_FWE-corr_ = 0.006 for right fusiform); and the small-volume-corrected result in mPFC also remained statistically significant (x = -2, y = 56, z = 15; T = 4.01; Z = 3.51; *P*_FWE-corr_ = 0.036). These small changes in statistical significance likely relate to the reduction in sample size resultant from removing four participants. The overall pattern of behavioural and imaging results is highly consistent, however.

Given the lack of difference between the faster-t2e subjects and the rest of the experimental sample both in terms of response time distributions and in terms of main effect of dominance and trustworthiness; the fact that these individuals are not at the extremes of the dominance-related slowing or untrustworthiness-related slowing distributions; and the fact that removing them from our analysis does not alter the pattern of our findings, we have retained all 36 participants in our analyses.

**Linearity and outliers in the correlations between GM volume and behavioral measures**

To investigate whether any of the reported correlations between dominance-related slowing and untrustworthiness-related slowing with focal GM volume could have been driven by statistical outliers in the data, we also examined scatterplots for each correlation (Figure S4 and Figure S5). We note that other than checking that our correlations are driven by linear relationships rather than outliers, these scatterplots cannot be interpreted further, as to do so would constitute a non-independent analysis and be an example of double-dipping (Kriegeskorte et al., 2009; Vul et al., 2009). There is an outlier in the scatterplot for the correlation between frontal opercular GM volume and dominance-related slowing (Figure S4). After removal of this outlier from analysis, this correlation is no longer significant after correction for multiple comparisons across the whole brain volume. However, small-volume correction at our *a priori* prediction of right insula (centred at a coordinate reported by Chiao et al., 2008; x = 39, y = 9, z = 15; see Materials and Methods section) results in a significant result at the same locus (x = 50, y = 2, z =13; T = 4.38, P_FWE-corr_ = 0.014) as our original whole-brain-corrected result. None of the other scatterplots (Figure S5 a-d) contain outliers. Given the right frontal opercular result is robust to outlier removal, and the remainder of our findings are not affected by outliers, we have retained all 36 subjects in our analysis.

**Are the structural correlates of preconscious dominance and untrustworthiness evaluation dissociable?**

In addressing this question, it is important to note that the behavioral measures of dominance-related slowing and untrustworthiness-related slowing are not correlated (see Behavioral results: Facial dominance and trustworthiness affect time-to-emergence). Moreover, when performing VBM analysis, we placed both dominance-related slowing and untrustworthiness-related slowing in the same SPM design matrix. Therefore, when VBM analysis was performed to explore GM-volume correlates of variability in one of these behavioral measures, the influence of the other behavioral measure was effectively regressed out since the multiple regression analysis performed was based on partial correlation. This makes GM volume findings related to variability in one behavioral measure unlikely to be strongly influenced by variability in the other behavioral measure.

We performed two additional analyses to test for dissociation in the structural correlates of dominance-related slowing and untrustworthiness-related slowing. Firstly, we explored whether there was any correlation between dominance-related slowing and GM volume at loci showing correlation with untrustworthiness-related slowing in our main results (pTPJ, fusiform gyrus and mPFC). Conversely, we also explored whether GM volume in right frontal operculum was correlated with untrustworthiness-related slowing. We performed these analyses by undertaking small-volume correction in a 15mm-radius sphere (or 8 mm-radius sphere in the case of fusiform gyrus) centred at the peak coordinate for each of our findings, setting a lenient threshold of p < 0.05, uncorrected. For dominance-related slowing there was a weak correlation (P_FWE-corr_ = 0.31) at x = 48, y = -60, z = 34, which is close to the TPJ locus we report at x = 51, y = -57, z = 31, as well as a weak correlation (P_FWE-corr_ = 0.29) at x = 41, y = -48, z = -17, near to our right fusiform result at x = 44, y = -46, z = -22. There were no suprathreshold correlations with GM volume in mPFC or left fusiform gyrus even at this lenient threshold. For untrustworthiness-related slowing, there was a weak positive correlation with GM volume in right frontal operculum (P_FWE-corr_ = 0.30) at x = 56, y = -9, z = 21, and an even weaker negative correlation (P_FWE-corr_ = 0.68) at x = 42, y = 2, z = 3. Both of these loci are some distance from the frontal opercular locus we report at x = 48, y = 2, z = 13.

For our second analysis to test for dissociation between the structural brain correlates of dominance-related slowing and untrustworthiness-related slowing, we extracted fitted responses from peak coordinates for our findings in right frontal operculum, right pTPJ, mPFC, right fusiform gyrus, and left fusiform gyrus, and calculated correlation coefficients between these and both dominance-related slowing and untrustworthiness-related slowing. For each region, we calculated the difference between the correlation with dominance-related slowing and the correlation with untrustworthiness-related slowing using Steiger’s Z-test (Steiger, 1980). For right frontal operculum, we found that the correlation with dominance-related slowing was significantly larger than the correlation with untrustworthiness-related slowing (r = -0.76 and r = 0.076, Z_1_bar = 4.27, p < 0.001). For the remaining four regions, we found that the correlation with untrustworthiness-related slowing was significantly larger than the correlation with dominance-related slowing (for pTPJ r = -0.76 and r = -0.076, Z_1_bar = -3.59, p < 0.001; for mPFC r = 0.55 and r = -0.055, Z_1_bar = 2.64, p = 0.008; for right fusiform r = -0.54 and r = -0.054, Z_1_bar = -2.80, p = 0.009; and for left fusiform r = -0.56 and r = -0.056, Z_1_bar = -2.21, p = 0.027).

These results rule out the possibility that GM volume in regions correlated with one of the behavioural measures was also significantly correlated with the other, and furthermore suggest that in all cases the correlation with one behavioral measure was significantly different to the correlation with the other. These findings support our claim of partial dissociability between the structural correlates of individual differences in dominance-related slowing and untrustworthiness-related slowing.

**Structural MRI correlates of individual differences in time-to-emergence and experimental task error**

We performed two additional VBM analyses. In both cases, a single measure was placed as the only regressor of interest in the SPM design matrix. In both cases we sought correlations with local GM that were significant after correction for multiple comparisons across the whole brain volume, or after small-volume correction using the same methodology and regions of interest (including bilateral amygdala and bilateral fusiform gyrus) as described in the Methods section. The first of these analyses found no correlations between individual differences in mean t2e (collapsed across all levels of facial dominance and trustworthiness) and local GM volume (both with whole-brain correction and with small-volume correction). The second of these analyses found no correlations between individual error rate (% of experimental trials where an incorrect response was given) and local GM volume after correction for multiple comparisons across the whole brain volume. However, with small-volume correction at our regions of interest, we found a significant correlation between error rate and GM volume at a locus (x = 44, y = 2, z = 16, P_FWE-corr_ = 0.032) found within a 15mm-radius sphere centred at coordinates for right insula (reported by Chiao et al., 2008; x = 39, y = 9, z = 15; and by Winston et al., 2002; x = 42, y = -4, z = 12). Error rate was not correlated with small-volume-corrected GM in TPJ, mPFC, fusiform gyrus, or amygdala.

**Relationship between amygdala volume and behavioral measures**

We wished to explore even more thoroughly the possibility of any relationship between amygdala structure and our behavioral measures. As an additional method for this, independent of our VBM analysis, we calculated amygdala volume for all 36 participants using automated subcortical segmentation, as described in Supplementary Methods. There was no correlation between amygdala volume and dominance-related slowing: for left amygdala [r = 0.11, *P* = 0.5]; for right amygdala [r = -0.04, *P* = 0.8]. There was also no correlation between amygdala volume and untrustworthiness-related slowing: for left amygdala [r = 0.02, *P* = 0.9]; for right amygdala [r = -0.001, *P* = 0.995]. Furthermore, we performed multiple regression analyses with dominance-related slowing or untrustworthiness-related slowing as dependent variables and age, gender identity, whole brain volume and either left or right amygdala volume as independent variables. This replicated the set of variables included in our VBM analysis. Again, we found that amygdala volume did not correlate with either behavioral measure after inclusion of these other covariates. When predicting dominance-related slowing, neither left amygdala volume [Beta 0.13, *P* = 0.55] nor right amygdala volume [Beta -0.12, *P* = 0.64] were significant predictors; when predicting untrustworthiness-related slowing, again neither left amygdala volume [Beta -0.06, *P* = 0.79] nor right amygdala volume [Beta -0.16, *P* = 0.51] were significant predictors.

**Relationship between cortical thickness, surface area and volume and individual differences in behavioral measures**

We performed surface-based analyses to compute cortical thickness, cortical surface area and cortical volume, and explored whether focal variability in any of these measures was correlated with our behavioral indices of dominance-related slowing and untrustworthiness-related slowing. Following cluster-wise correction for multiple comparisons, we found no brain regions where cortical thickness, cortical surface area, or cortical volume was significantly correlated with dominance-related slowing or untrustworthiness-related slowing.

Supplementary Discussion

**Variation of statistical significance for the behavioral main effect of trustworthiness in different experimental samples**

The main effect of trustworthiness did not quite reach statistical significance in our behavioral results. However, this effect has previously been demonstrated to be significant in two separate and independent samples of participants (Stewart et al., 2012). While the pattern of our findings for the effect of trustworthiness on t2e is the same as that reported by Stewart et al., we would argue that the difference in significance is likely due to differences in participant personality traits in the different samples. Stewart et al. (2012) showed that individual untrustworthiness-related slowing is negatively correlated with self-rated scores on a Propensity to Trust Scale. It is thus quite possible that the present sample of participants contained more trusting individuals (i.e. individuals with higher scores on the self-rated scale measuring propensity to trust others) than the sample of Stewart et al., which would be reflected in smaller differences, at the group level, between t2e for least trustworthy and neutral-trustworthiness faces. We did not collect Propensity to Trust scores from our participants and are thus unable to fully verify this possibility. However, given both previous and current findings, we are confident that there is an effect of face trustworthiness on t2e that is highly variable between individuals, and this, rather than the group-level main effect, is the key observation explored in our VBM analysis.

**Are the structural neural correlates of preconscious dominance and untrustworthiness evaluation dissociable?**

We have shown that individual dominance-related slowing and untrustworthiness-related slowing are not correlated (see Behavioral results: facial dominance and trustworthiness affect t2e). Our VBM analysis was designed in such a way that when testing for correlations between gray matter volume and one of these behavioral measures, the influence of the other behavioral measure was effectively regressed out. Furthermore, we have shown that there was no correlation between dominance-related slowing and GM volume at loci showing correlation with untrustworthiness-related slowing, nor was there any correlation between untrustworthiness-related slowing and GM volume at loci showing correlation with dominance-related slowing. Finally, by extracting GM volume values from peak results in our regions of interest, we can show that in the case of every region, the correlation with one behavioral measure is significantly different to that with the other behavioral measure. These observations provide support for our claim that there are at least partially separable neuronal substrates for preconscious evaluation of dominance and trustworthiness.

**Structural neural correlates of individual differences in task error rate**

The result of our VBM analysis of correlations between behavioral experimental error rate and local GM volume might lead one to speculate that our finding of a correlation between dominance-related slowing and GM in right frontal operculum in fact reflects individual differences in error rate, or otherwise ability to appropriately perform the task irrespective of social dominance evaluation. We would argue that this is unlikely since error rate does not correlate significantly or meaningfully with our measures of dominance or trustworthiness evaluation. We therefore think it more likely that GM volume in two adjacent regions in right frontal operculum correlates with preconscious social dominance evaluation and with error rate on a left/right task (the latter finding may well relate to individual differences in response inhibition, which would fit with known functional roles of nearby IFG; e.g. Aron et al., 2004). Error rate was not correlated with small-volume-corrected gray matter in TPJ, mPFC, fusiform gyrus, or amygdala.

Supplementary References

Aron, A.R., Robbins, T.W., Poldrack, R.A. (2004) Inhibition and the right inferior frontal cortex. *Trends in Cognitive Sciences, 8*, 170–177.

Chiao, J.Y., Adams, R.B., Tse, P.U., Lowenthal, L., Richeson, J.A., Ambady, N. (2008) Knowing who’s boss: fMRI and ERP investigations of social dominance perception. *Group Processes & Intergroup Relations*, *11*, 201–214.

Fischl, B., Salat, D.H., Busa, E., Albert, M., Dieterich, M., Haselgrove, C., et al. (2002) Whole brain segmentation: automated labeling of neuroanatomical structures in the human brain. *Neuron, 33*, 341–355.

Fischl, B., & Dale, A.M. (2000) Measuring the thickness of the human cerebral cortex from magnetic resonance images. *Proceedings of the National Academy of Sciences of the USA,* *97*, 11050-11055.

Fischl, B., Sereno, M.I., Dale, A.M. (1999) Cortical surface-based analysis. II: Inflation, flattening and a surface-based coordinate system. *Neuroimage, 9*, 195-207.

Hagler Jr., D.J., Saygin, A.P., Sereno, M.I. (2006) Smoothing and cluster thresholding for cortical surface-based group analysis of fMRI data. *Neuroimage 33*, 1093–1103.

Hutton, C., Draganski, B., Ashburner, J., Weiskopf, N. (2009) A comparison between voxel-based cortical thickness and voxel-based morphometry in normal aging. *Neuroimage, 48*, 371–380.

Kriegeskorte, N., Simmons, W.K., Bellgowan, P.S.F., Baker, C.I. (2009) Circular analysis in systems neuroscience: the dangers of double dipping. *Nature Neuroscience, 12*, 535–540.

Steiger, J.H. (1980) Tests for comparing elements of a correlation matrix. *Psychological Bulletin 87*, 245–251.

Stewart, L.H., Ajina, S., Getov, S., Bahrami, B., Todorov, A., Rees, G. (2012) Unconscious evaluation of faces on social dimensions. *Journal of Experimental Psychology: General,141*, 715–727.

Vul, E., Harris, C., Winkielman, P., Pashler, H. (2009) Puzzlingly high correlations in fMRI studies of emotion, personality, and social cognition. *Perspectives on Psychological Science, 4*, 274–290.

Winston, J.S., Strange, B.A., O’Doherty, J., Dolan, R.J. (2002) Automatic and intentional brain responses during evaluation of trustworthiness of faces. *Nature Neuroscience, 5*, 277–283.
